# Secondary Traumatic Stress of Interpreters Working in Psychotherapy: Protective, Risk and Interpreter‐Specific Factors

**DOI:** 10.1002/cpp.70305

**Published:** 2026-07-07

**Authors:** Monja Lucia Herold, Bianca Schreyer, Rita Rosner

**Affiliations:** ^1^ Department of Psychology Catholic University of Eichstätt‐Ingolstadt Eichstätt Germany

**Keywords:** interpreter, language mediator, mental health, refugee, secondary traumatic stress, working conditions

## Abstract

**Background:**

Interpreters working in psychotherapy are frequently exposed to emotionally demanding situations and may develop secondary traumatic stress. However, quantitative research has largely focused on community interpreters, highlighting the need for data on symptom levels and associated risk factors among interpreters in psychotherapeutic settings.

**Methods:**

Sixty‐three eligible interpreters were included. Secondary traumatization, psychological distress, resources, competence, occupational distress, empathy, as well as interpreter‐specific aspects such as flight experience and identification with the patient's cultural background were assessed using validated instruments. Data were analyzed using correlations and hierarchical multiple regression to examine general and interpreter‐specific risk factors.

**Results:**

14.29% of participants scored above the clinical cutoff for secondary traumatic stress. In a hierarchical regression model predicting secondary traumatic stress (STS), the final model explained a substantial proportion of variance (adjusted *R* ^2^ = 0.52). In Step 3, interpreter‐specific factors contributed significantly to the model (Δ*R* ^2^ = 0.25, F_change (2,48) = 14.10, *p* < 0.001), with flight experience showing a significant negative association with STS (*β* = −0.54, 95% CI [−0.97, −0.16]) and identification with the patient's cultural background showing a significant positive association (*β* = 0.27, 95% CI [0.05, 0.47]).

**Conclusion:**

Interpreters with personal flight experience and strong identification with the client's cultural background appear particularly vulnerable to secondary traumatic stress. Psychological distress, resources, competence, occupational distress, and empathy did not influence secondary traumatic stress. This suggests that support such as supervision and training should focus more specifically on interpreters' personal experiences and cultural match.

## Summary

- Approximately 14% of interpreters working in psychotherapy reported clinically relevant secondary traumatic stress.
- Strong identification with a client's cultural background was associated with higher levels of secondary traumatic stress.
- Supervision and training should address interpreters' personal experiences and cultural identification with clients.

## 1 Introduction

Interpreters play an important role in psychotherapy, facilitating effective communication when linguistic differences between the psychotherapist and the patient impede it (Fennig and Denov 2021; Geiling et al. 2021). There are no standardized trainings or obligatory supervision for community interpreters, including those working in psychotherapy, and little is known about their actual working conditions (Hanft‐Robert and Mösko 2024). As professional interpreters are unavailable for many languages required in psychotherapy with refugees, ad hoc interpreters—often refugees themselves—are frequently recruited for this demanding task. These interpreters often share similar experiences with clients, including flight experiences, cultural background and potential collective traumas (Miller et al. 2005).

Trauma‐focused cognitive behavioural therapy presents interpreters with the challenge of accurately relaying patients' traumatic experiences in their exact words and from their perspective, which is particularly demanding (Kießl et al. 2017; Lai and Costello 2021; Villalobos et al. 2021). Qualitative studies describe stress and overwhelming emotions among interpreters, especially when patients' experiences resonate with their own (Fennig and Denov 2021; Geiling et al. 2021; Simms et al. 2021). The distress caused by exposure to traumatic material in professionals is referred to as secondary traumatic stress (STS) (Figley 1995), a concept also applied to interpreters (Hapfelmeier et al. 2025; Kindermann et al. 2017). Quantitative studies show that interpreters often struggle to disengage after challenging sessions, reporting intrusive thoughts and symptoms such as nightmares, sleep disturbances, irritability and persistent preoccupation (Yick and Daines 2019). These reactions may also affect the therapeutic process through avoidance, reduced empathy or emotional overwhelm that interrupts sessions and negatively impacts the quality of interpretation (Lai and Heydon 2015; Miller et al. 2005; Simms et al. 2021).

Psychological distress, including personal trauma history, must be considered as a potential cause of STS among community interpreters (Fennig and Denov 2021). Studies report that approximately 10% of interpreters working with refugees show indications of posttraumatic stress disorder (PTSD) (Kindermann et al. 2017; Teegen and Gönnenwein 2002). In addition, symptoms of depression and anxiety appear to be more prevalent among community interpreters than in the general population and may contribute to STS (Geiling et al. 2021; Hanft‐Robert and Mösko 2024).

Research on STS in psychotherapy identifies several protective and risk factors, including individual resources, professional competence, occupational distress and empathy. Among individual resources, Antonovsky's sense of coherence (SOC) is considered an important salutogenic factor associated with lower psychological strain, including STS, across helping professions (Greinacher et al. 2019; Steinlin et al. 2017). The ‘sense of coherence’ (SOC), introduced by Aaron Antonovsky, describes a person's enduring confidence that life is comprehensible, manageable and meaningful. According to Antonovsky, people with a strong SOC are better able to cope with stress and maintain their health. (Moksnes 2021). Evidence for interpreters remains limited; however, Kindermann et al. (2017) found strong negative associations between SOC and STS in community interpreters. Social support represents another well‐established protective factor shown to reduce stress symptoms and secondary traumatization across diverse samples (Galek et al. 2011; Hensel et al. 2015; MacRitchie and Leibowitz 2010). Together, SOC and social support can be understood as core individual resources protecting against STS.

Professional competence may also function as a protective factor. Baird and Jenkins (2003) showed that role competence, including task knowledge and problem‐solving skills, was associated with lower STS among domestic violence therapists, while higher education and clinical exposure were linked to less vicarious trauma. Similarly, trauma‐related training and professional preparation were associated with lower STS among school counsellors (Rumsey et al. 2020). In qualitative research, interpreters frequently report knowledge gaps and insufficient preparation for their demanding work (Fennig and Denov 2021; Miller et al. 2005; Morina et al. 2010; Wichmann et al. 2018). Interpreters with formal qualifications report lower psychological strain (Geiling et al. 2021), though quantitative evidence remains scarce (Hanft‐Robert and Mösko 2024). While findings regarding professional experience are mixed, more experienced interpreters may be better able to manage job‐related challenges (Geiling et al. 2021). Overall, professional competence may help interpreters feel more secure in their role and thereby reduce vulnerability to STS.

Beyond individual competence, work‐related factors may also influence STS in mental health professionals. Access to supervision has been associated with lower STS (Fernández et al. 2024; Quinn et al. 2019), while organizational support and manageable workloads are considered protective influences in trauma‐exposed professions (Devilly et al. 2009; Geiling et al. 2022). Among interpreters working with refugees, occupational stressors such as low income, high workload, and limited professional support have been identified as potential risks for STS (Denkinger et al. 2018; Hanft‐Robert and Mösko 2024). Regular supervision, in contrast, appears to mitigate stress (Geiling et al. 2021; Miller et al. 2005). Taken together, insufficient compensation, extensive workload and lack of professional support represent occupational distress that requires further quantitative investigation.

Empathy, a key component of perspective‐taking and engagement (Maes et al. 1995), has also been discussed as a potential risk factor. Findings among psychotherapists are inconsistent, with some studies reporting positive associations between empathy and STS (Püttker et al. 2015) and others finding no relationship (Bridger et al. 2020). Further research is needed to clarify the role of empathy in interpreters working in psychotherapy.

In addition to these general factors, interpreters in community and psychotherapeutic settings often work in ad hoc roles. Specific risks compared to other helping professions may arise from their higher likelihood of personal flight experiences (Kindermann et al. 2017; Miller et al. 2005). Qualitative research highlights that identification with clients beyond empathy can increase emotional strain and vulnerability to STS (Green et al. 2012; Mehus and Becher 2016; Splevins et al. 2010). More empirical evidence on the influence of shared cultural background is therefore needed.

The present study addresses the lack of empirical data on interpreters working in psychotherapy by examining STS and its associations with general risk factors, including psychological and occupational distress, as well as individual resources, professional competence and empathy. While some aspects have been examined separately in the limited empirical literature, they have not yet been investigated jointly. In addition, interpreter‐specific factors such as personal flight experience and identification with the patient's cultural background are considered.

## 2 Method

### 2.1 Recruitment

The sample was recruited nationwide in Germany by identifying interpreter pools and relevant institutions (e.g., psychosocial centres) and compiling them into a contact list. Institutions were contacted by phone or email, and the survey was distributed to 102 institutions. Additional distribution occurred via professional networks, including the Ethno‐Medical Center's mailing list (> 500 potential participants) and the internal member portal of the Federal Association of Interpreters and Translators. The initially intended inclusion criterion of experience in trauma‐focused psychotherapy could not be fully verified because of inconsistent responses to a control question on trauma‐specific knowledge. Therefore, participation in psychotherapy in general was used as the inclusion criterion. In total, 201 individuals were assessed for eligibility; 190 provided informed consent. After exclusions due to noneligibility, dropout and missing data, 124 participants remained eligible for analysis. Of the participants who met eligibility criteria, 60 discontinued the survey before completion and were therefore not included in the final analyses because they did not complete the primary outcome measure (FST), which was administered toward the end of the questionnaire. As most discontinuations occurred in later sections of the survey, attrition may have been related to survey length or fatigue. Comparisons based on the available demographic variables did not indicate meaningful differences between participants who discontinued after eligibility screening and those who completed the survey.

**FIGURE 1:**
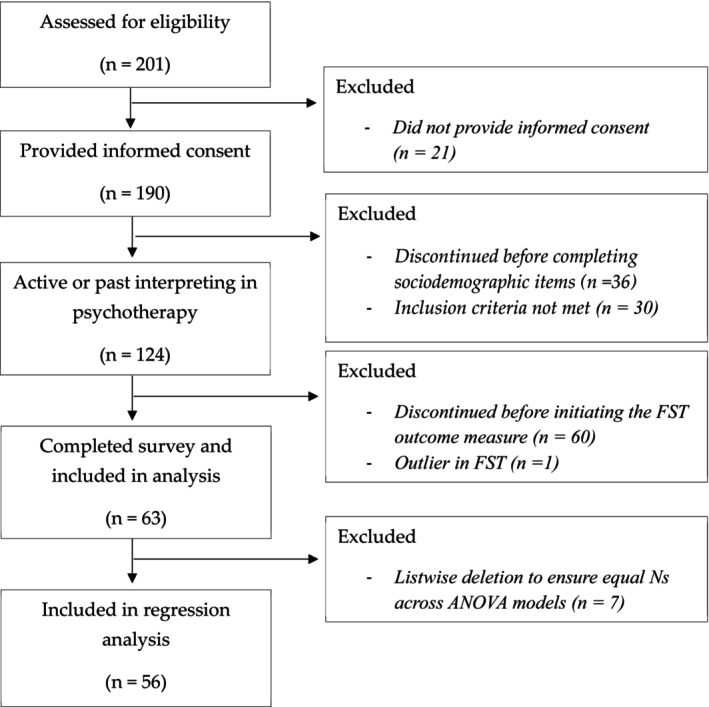
Diagram of participants flow.

Additionally, seven completed cases were excluded from the final analytic sample due to substantial missing, implausible, and/or outlier data patterns; thus, 56 were retained for regression analyses following listwise deletion (see Figure 1).

### 2.2 Sample

Of the participants, 74.6% identified as female (*n* = 47) and 25.4% as male (*n* = 16) (see Table 1). Participants were between 25 and 75 years old (M = 45.00, SD = 12.21). Educational levels ranged from no school‐leaving qualification to university entrance qualification, the latter being the most common (55.6%, *n* = 35). A migration background was reported by 85.7% of respondents (*n* = 54). Participants represented diverse national backgrounds, with the largest groups originating from Afghanistan, Iran, Syria, Russia, Ukraine and Turkey, while all other countries were represented by one or two individuals. The linguistic background was highly diverse, with over 30 reported languages. The most frequent working languages included Russian, French, Arabic, Dari, Farsi/Persian, English, Kurdish and Spanish. Many interpreters reported working with multiple language combinations.

**TABLE 1:** demographic characteristics of the interpreters (*n* = 63).

Characteristics	*n*	%
Gender		
Female	47	74.6
Male	16	25.4
Age (years)		
18–29 years	5	7.9
30–39 years	18	18.6
40–49 years	21	33.3
50–59 years	9	14.3
60 years and older	10	15.9
FST (≥ 65)	11	17.3
Psychological distress		
PTSD (≥ 33)	29	50.9
PHQ‐2 (≥ 3)	12	19.7
GAD‐2 (≥ 3)	8	13.1
Education		
No school‐leaving qualification	1	1.6
(Qualified) lower secondary school certificate	5	7.9
Intermediate secondary school certificate	9	14.3
University of applied sciences entrance qualification	13	20.6
General university entrance qualification	35	55.6
Migration background	54	85.7
German language level		
B1	3	4.8
B2	7	11.1
C1	9	14.3
C2	31	49.2
Country of origin		
Afghanistan	7	12.9
Iran	5	9.3
Syria	5	9.3
Russia	5	9.3
Ukraine	4	7.4
Turkey	3	5.6
Other countries	34	55.6
Translated language		
Russian	14	—
French	10	—
Arabic	8	—
Dari	8	—
Farsi/Persian	8	—
English	8	—
Kurdish	6	—
Spanish	6	—
Other languages	≤ 3 each	—

*Note:* Other countries of origin included Albania, Algeria, Armenia, Belarus, Belgium, Brazil, Bulgaria, Chile, China, Djibouti, Eritrea, France, Ghana, Guinea, India, Iraq, Italy, Yugoslavia, Kazakhstan, Kyrgyzstan, Colombia, Croatia, Kurdistan, North Macedonia, Morocco, Mexico, Montenegro, Pakistan, Palestine, the Philippines, Poland, Romania, Russia, Senegal, Serbia, Spain, Tajikistan, Togo, Tunisia, Ukraine, and the. Other languages included Arabic, Armenian, Bosnian, Bulgarian, Croatian, Dari, English, Farsi/Persian, French, German, Ingush, Italian, Kurdish, Macedonian, Pashto, Portuguese, Punjabi, Romanian, Russian, Serbian, Serbo‐Croatian, Spanish, Ukrainian, Urdu, and Creole varieties; participants could report multiple languages; therefore, no percentages were calculated.

Abbreviations: FST, Fragebogen zur sekundären Traumatisierung (Secondary Traumatization Questionnaire); GAD‐2, Generalized Anxiety Disorder 2‐item; PCL‐5, PTSD Checklist for DSM‐5; PHQ‐2, Patient Health Questionnaire 2‐item.

### 2.3 Measurements

#### 2.3.1 Sociodemographic Data

Sociodemographic information included gender, age, highest educational attainment, migration background and country of origin. Participants also reported personal flight experience, mother tongue and German language proficiency, assessed using CEFR level codes. To obtain detailed information on interpreting activities, participants reported their interpreter training. Given the heterogeneity of professional backgrounds in psychotherapy interpreting, the following four categories were provided: no specific training, workshops (up to four weekends), training (more than four weekends) and university degree. Interpreted languages were recorded via an open‐response format. As current or previous interpreting in therapy was an inclusion criterion, participants indicated whether they were interpreting at the time of the study or had done so in the past.

#### 2.3.2 Secondary Traumatization

Secondary traumatic stress was assessed using the acute version of the Secondary Traumatization Questionnaire (FST; Daniels 2006), a 31‐item measure rated on a five‐point scale (1 = *never* to 5 = *very often*) referring to symptoms during the previous week. Scores ≥ 65 indicate moderate STS and scores > 82 severe STS. Validation studies showed excellent internal consistency (*α* = 0.92–0.94; Weitkamp et al. 2014). Internal consistency in the present sample was also excellent (*α* = 0.94) (Table 2).

**TABLE 2:** Descriptive statistics of parameters (*n* = 63).

Parameter	%/M	*n*/SD
FST (M/SD)	49.48	15.74
Psychological distress		
PCL‐5 (M/SD)	38.3	16.99
PHQ‐2 (M/SD)	1.62	1.34
GAD‐2 (M/SD)	1.41	1.32
Resources		
SOC‐L9 (M/SD)	28.9	9.71
F‐SozU K14 (M/SD)	54.3	11.83
Competence		
Knowledge (M/SD)	2.86	1.17
Experience in years (M/SD)	9.87	9.06
No specific training (%/*n*)	31.75	20
Workshop (up to four weekends) (%/*n*)	19.05	12
Training program (more than four weekends) (%/*n*)	20.63	13
University degree (%/*n*)	28.57	18
Occupational distress		
Interpreting hours per week (M/SD)	10.54	9.71
Monthly net income in € (M/SD)	948.11	1227.98
Offered supervision (%/*n*)	50.79	32
Hours of supervision last 6 months (M/SD)	1.45	0.76
Relationship with therapist range 1–5 (M/SD)	4.05	0.96
Respect by therapist range 1–5 (M/SD)	4.19	0.72
EMPT7 (M/SD)	32.57	6.14
Flight experience (%/*n*)	33.3	21
Identification with cultural background (M/SD)	2.71	0.96

*Note:* FST, Fragebogen zur sekundären Traumatisierung (Secondary Traumatization Questionnaire); EMPT7, Empathy and Perspective‐Taking Questionnaire; F‐SozU K14, Fragebogen zur sozialen Unterstützung (Questionnaire for social support); GAD‐2, Generalized Anxiety Disorder 2‐item; PCL‐5, PTSD Checklist for DSM‐5; PHQ‐2, patient health questionnaire 2‐item; SOC‐L9, Sense of Coherence 9‐item Leipzig Short Scale.

#### 2.3.3 Psychological Distress

PTSD symptoms were assessed using the PTSD Checklist for DSM‐5 (PCL‐5; Weathers et al. 2013). Exposure to Criterion A events was screened using the LEC‐5. Symptoms during the past month were rated on a 0–4 scale, with scores ≥ 33 indicating probable PTSD (Krüger‐Gottschalk et al. 2017). Internal consistency was excellent (*α* = 0.96). Depressive and anxiety symptoms were assessed using the PHQ‐2 (Kroenke et al. 2003; Löwe et al. 2005) and GAD‐2 (Hinz et al. 2017; Plummer et al. 2016), with cutoffs ≥ 3 indicating elevated symptoms. Internal consistencies were acceptable (PHQ‐2 *α* = 0.68; GAD‐2 *α* = 0.66). For analyses, PCL‐5, PHQ‐2 and GAD‐2 scores were combined into a composite index of psychological distress (*α* = 0.95).

#### 2.3.4 Resources

The SOC was assessed using the nine‐item Leipzig Short Scale (SOC‐L9; (Schumacher et al. 2000)). This scale was rated on seven‐point semantic differentials, and scores were recoded so that higher scores reflected stronger SOC (range 9–63). The internal consistency of the scale was satisfactory (*α* = 0.87). The 14‐item *Fragebogen zur Sozialen Unterstützung* (F‐SozU K14; (Fydrich et al. 2009)) was used to assess perceived social support and demonstrated excellent reliability (*α* = 0.94). Both scales were *z*‐standardized and combined into a composite indicator of personal resources, which demonstrated excellent internal consistency (*α* = 0.94).

#### 2.3.5 Competence

PTSD knowledge was measured with four multiple‐choice items from the TF‐CBT training manual (Heck et al. 2015), each offering one correct answer (0–4 points). The items covered included symptomatology, epidemiology/terminology, aetiology and diagnostics. Two additional indicators were used to assess interpreters' expertise: years of interpreting experience in therapeutic settings and professional interpreter qualification. All three variables were *z*‐standardized and combined into a composite competence score.

#### 2.3.6 Occupational Stress

Work‐related conditions were measured using individually developed items assessing key aspects of workload, including low income, long weekly working hours and limited professional support (e.g., supervision availability, perceived collaboration with therapists). Response formats varied (open‐ended, categorical and Likert‐type). All variables were *z*‐standardized and combined into a composite score representing occupational stress.

#### 2.3.7 Empathy

Empathy was measured using the nine‐item Empathy and Perspective‐Taking Questionnaire (EMPT7) by Maes and Schmitt, M. and Schmal, A. (1995), based on the Empathic Concern and Perspective Taking subscales of the Interpersonal Reactivity Index (Davis and Davis 1983). Items were rated on a five‐point scale (0–4), yielding scores from 0 to 36, with higher values indicating greater empathy. Internal consistency in the present sample was high (*α* = 0.87).

#### 2.3.8 Flight Experience

Interpreters were additionally asked about personal refugee experience using a dichotomous item (1 = *no refugee experience*, 0 = *refugee experience*).

#### 2.3.9 Identification With Cultural Background

Identification with clients was assessed using a single item (‘How strongly do you identify with clients who share your cultural background?’) rated on a five‐point Likert scale (1 = *not at all* to 5 = *very strongly*).

### 2.4 Data Analysis

Analyses were conducted in R Studio (Version 2024.12.1) (R Core Team 2024) using the packages {Hmisc} (Harrell 2024) for correlation analyses, {lmtest} (Zeileis and Hothorn 2002) or heteroscedasticity testing and {car} (Fox and Weisberg 2019) for multicollinearity diagnostics and bootstrapping. Prior to analysis, all variables were *z*‐standardized, and theoretically derived composite scores were computed to represent the constructs of psychological distress (symptoms of posttraumatic stress, depression and anxiety), occupational distress (salary, working hours and professional support), competence (knowledge test and years of experience) and resources (social support and SOC). One outlier with an FST‐score of |*z*| > 4 was excluded from the analysis. We computed Pearson's correlation coefficients *r* before and after calculating composite scores. Subsequently, a hierarchical multiple regression was conducted to predict secondary traumatic stress. The predictors were entered in three conceptually defined steps: first psychological distress, second general protective and risk factors (occupational distress, resources, competence and empathy); then, interpreter‐specific risk factors (flight experience and identification). Subsequently, a theory‐driven hierarchical multiple regression was conducted to predict secondary traumatic stress. Predictors were entered sequentially in conceptually defined blocks: (1) psychological distress variables, (2) general occupational and protective factors (occupational distress, resources, competence and empathy) and (3) interpreter‐specific variables (flight experience and identification with the patient's cultural background). Nested models were compared using changes in explained variance (Δ*R* ^2^), hierarchical *F*‐tests and information criteria (AIC and BIC) to evaluate the incremental contribution of each predictor block. Scatter plots were used to confirm the linear relationship between the variables. According to Q‐Q plots and the Shapiro–Wilk test, residuals were normally distributed. As evidence of heteroscedasticity was found, nonparametric adjustments to the standard errors and confidence intervals were made using bootstrapping (Chernick 2007), with 5000 resamples. The variance inflation factor (VIF) was used to investigate collinearity in each model, and no collinearity was detected (i.e., VIF < 5 for all predictors). Tests of regression assumptions can be found in Appendix A. All tests were two‐tailed with an α‐level of 0.05.

## 3 Results

### 3.1 Parameters of Predictors

Based on the recommended cutoff of 33 for probable PTSD, 29 participants (50.9%) screened positively. For secondary traumatic stress (STS), *n* = 11 participants (17.4%) scored above the clinical cutoff of 65 and *n* = 4 (4.8%) participants scored above the cutoff of ≥ 85 for severe STS. Using the established cutoff of ≥ 3 for elevated depressive symptoms, 19.7% of participants (*n* = 12) scored above the threshold with the PHQ‐2 and for GAD‐2, 13.11% of participants (*n* = 8) scored above the threshold (see Table 1).

Secondary stress showed a mean of M = 49.48 (SD = 15.74) (see Table 2). Psychological distress indicators yielded M = 38.30 (SD = 16.99) on the PCL‐5, M = 1.62 (SD = 1.34) on the PHQ‐2 and M = 1.41 (SD = 1.32) on the GAD‐2. SOC (1–7 scale, nine items) averaged M = 28.90 (SD = 9.71), and perceived social support (1–5 scale) averaged M = 54.32 (SD = 11.83). Competence indicators showed mean knowledge scores of M = 2.86 (SD = 1.17; 0–5 scale), an average of M = 9.87 years of professional experience (SD = 9.06), and 50.8% of participants held an interpreter qualification. Work‐related conditions included M = 10.54 weekly interpreting hours (SD = 9.71) and a mean monthly net income of M = €948.11 (SD = €1227.98). Half of the sample (50.8%) reported access to supervision, and those receiving it reported M = 1.45 h within the past 6 months (SD = 0.76). Relationship quality with therapists was rated at M = 4.05 (SD = 0.96; 1–5 scale) and perceived respect at M = 4.19 (SD = 0.72; 1–5 scale). Empathy scores averaged M = 32.57 (SD = 6.14), 33.3% reported personal flight experience, and cultural identification was M = 2.71 (SD = 0.96; 1–5 scale).

### 3.2 Correlations

Table 3 presents the correlation matrix of all predictors included in the multiple regression analysis. STS was positively and strongly correlated with psychological distress (*r* = 0.83, *p* < 0.05) and identity‐related factors (*r* = 0.82, *p* < 0.05). In contrast, resources (*r* = −0.86, *p* < 0.01) and flight experience (*r* = −0.83, *p* < 0.05) showed strong negative associations with STS. All other correlations between STS and occupational distress, competence and empathy were nonsignificant. Among the predictors, a strong negative association was also observed between psychological distress and resources (*r* = −0.97, *p* < 0.01). No other correlations reached statistical significance. The correlations of the individual variables, without aggregation into composite scores, are presented in Appendix B. Due to the very high correlation between psychological distress and resources (r = 0.97), additional sensitivity analyses were conducted excluding each variable separately from the regression model. Results indicated a suppression effect: Psychological distress became statistically significant when resources were excluded, whereas resources became statistically significant when psychological distress was excluded. The remaining pattern of results, particularly for flight experience and identification, remained largely stable across models (Appendix C).

**TABLE 3:** Correlation matrix of predictors included in the multiple regression.

Variable	1	2	3	4	5	6	7
1. Secondary stress							
2. Psychological distress	0.83*						
	[0.31, 0.97]						
3. Occupational distress	0.18	0.09					
	[−0.60, 0.78]	[−0.66, 0.75]					
4. Resources	−0.86**	−0.97**	−0.25				
	[−0.97, −0.38]	[−1.00, −0.85]	[−0.81, 0.55]				
5. Competence	−0.28	−0.14	−0.53	0.22			
	[−0.82, 0.53]	[−0.77, 0.63]	[−0.90, 0.28]	[−0.58, 0.80]			
6. Empathy	−0.12	−0.06	−0.62	0.17	0.45		
	[−0.76, 0.64]	[−0.74, 0.67]	[−0.92, 0.15]	[−0.61, 0.78]	[−0.38, 0.88]		
7. no Flight experience	−0.83*	−0.51	−0.42	0.54	0.45	0.24	
	[−0.97, −0.29]	[−0.89, 0.30]	[−0.87, 0.41]	[−0.27, 0.90]	[−0.37, 0.88]	[−0.56, 0.81]	
8. Identity	0.82*	0.68	−0.14	−0.65	−0.21	0.14	−0.67
	[0.28, 0.97]	[−0.06, 0.94]	[−0.77, 0.63]	[−0.93, 0.10]	[−0.80, 0.58]	[−0.62, 0.77]	[−0.93, 0.07]

*Note:* Values in square brackets indicate the 95% confidence interval for each correlation.

* *p* < 0.05.

** *p* < 0.01.

### 3.3 Hierarchical Regression Analyses

A hierarchical multiple regression analysis was conducted to examine the predictors of secondary traumatic stress across three successive models. Step 1 included psychological distress as the only predictor, explaining a substantial proportion of the variance, *R* ^2^ = 0.27, *F*(1,54) = 20.85, *p* < 0.001. Higher psychological distress was significantly associated with higher secondary traumatic stress, *β* = 0.51, 95% CI [0.31, 0.75], *p* < 0.001. Step 2 incorporated general protective and risk factors but accounted for no significantly greater proportion of variance than Step 3, Δ*R* ^2^ = 0.27, *F*(4,50) = 1.55, *p* = 0.20. None of the predictors in the second model reached statistical significance. In Step 3, we incorporated interpreter‐specific risk factors, including experience of flight and identification. There was a significant increase in explained variance in Step 3, Δ*R* ^2^ = 0.25, *F*(1, 49) = 14.10, *p* < 0.001. The third model also produced lower AIC and BIC scores than the first two models (Model 1: AIC = 118.86, BIC = 124.93; Model 2: AIC = 122.47, BIC = 136.65; Model 3: AIC = 100.59, BIC = 118.81). The only predictors contributing to the final model were experience of flight (*β* = −0.54, 95% CI [−0.97, −0.16], *p* < 0.01) and identification (*β* = 0.27, 95% CI [0.05, 0.47], *p* < 0.01), indicating higher levels of secondary traumatic stress for those who experienced flight and for those with a higher level of identification (Table 4).

**TABLE 4:** Hierarchical regression model predicting secondary traumatic stress.

Predictor variable	Step 3			Model		Step	
	*β*	Bootstrapped 95% CI	Bootstrapped SE *β*	*Adj. R* ^2^	*F*‐test	*ΔR* ^2^	*F*‐test (change)
Step 1							
Intercept	**0.30**	**0.01, 0.61**	0.15	0.27	*F*_1,54_ = 20.85	0.27	*F*_1, 54_ = 31.82
Psychological distress	0.24	−0.08, 0.47	0.14		*p* < 0.001		*p* < 0.001
Step 2							
Occupational distress	0.23	−0.33, 0.75	0.28	0.27	*F*_5,50_ = 4.99	0.05	*F*_4,50_ = 1.55
Resources	−0.15	−0.37, 0.12	0.13		*p* < 0.001		*p* = 0.20
Competence	0.17	−0.11, 0.50	0.15				
Empathy	0.19	−0.15, 0.18	0.08				
Step 3							
No flight experience	**−0.54** **	**−0.97, −0.16**	0.21	0.52	*F*_7,48_ = 9.46	0.25	*F*_2,48_ = 14.10
Identification	**0.27** **	**0.05, 0.47**	0.11		*p* < 0.001		*p* < 0.001

*Note:* Regression coefficients and confidence intervals where the 95% bootstrapped confidence interval did not include zero are highlighted in bold font.

* *p* < 0.05.

** *p* < 0.01.

## 4 Discussion

The present study examined general and interpreter‐specific risk factors for STS among interpreters working in psychotherapy. Overall, participants reported notable levels of STS, and hierarchical regression analyses showed that interpreter‐specific factors explained additional variance beyond general psychological and occupational factors. Flight experience was negatively associated with STS, whereas identification with the patient's cultural background was positively associated with STS, highlighting the relevance of context‐specific characteristics in this setting.

In the present sample, 17% of interpreters showed considerable STS and 4% severe STS. These rates are slightly lower than those reported in other quantitative studies of interpreters, which found prevalences of 21% (Kindermann et al. 2017) and 22% (Wichmann et al. 2018). Denkinger et al. (2018), who examined interpreters and other helping professionals, reported signs of secondary traumatization in 22.9% of their sample. Overall, the distribution of STS appears comparable to previous research.

### 4.1 Psychological Distress

The average PTSD symptom score suggested clinically relevant trauma‐related distress. Approximately half of the interpreters reported trauma‐related symptoms at a clinically relevant level, exceeding previously reported rates. For example, Kindermann et al. (2017) found elevated trauma symptoms in about 10% of interpreters. In contrast, mean depression and anxiety scores remained below screening thresholds, indicating generally subclinical symptoms. These rates also appear lower than those reported by Hanft‐Robert and Mösko (2024) among community interpreters. Overall, interpreters in this study experienced moderate to high psychological distress, highlighting considerable psychological strain within this professional group. Consistent with previous research (Fennig and Denov 2021; Kindermann et al. 2017; Wichmann et al. 2018), psychological distress was positively correlated with STS, indicating that higher distress was associated with higher STS. However, this association disappeared when additional general risk and protective factors were included in the second step of the regression analysis. This may reflect shared variance among predictors, leaving little unique variance for individual variables in the full model, a phenomenon well documented in regression research (Cohen et al. 2013). Psychological distress became statistically significant once resources were excluded from the model, suggesting substantial shared variance between both constructs. This pattern may indicate that psychological distress and perceived resources capture closely related aspects of participants' psychological functioning in the present sample. Additionally, the findings suggest that interpreter‐specific factors, such as identification with the patient's cultural background, may represent relevant correlates of mental health outcomes alongside psychological distress. However, given the high correlations among predictors, these variables may reflect overlapping variance rather than distinct independent contributions.

### 4.2 General Risk and Protective Factors

In line with earlier research, general risk and protective factors frequently discussed in the literature showed high correlations with STS but did not predict STS in the regression analysis. Although previous studies suggest that internal and external resources buffer secondary traumatization among interpreters (Kindermann et al. 2017), this effect could not be replicated. One explanation may be the substantial overlap between resources and psychological distress, which limits the unique contribution of resources in the regression model and suggests that resources may operate as moderating rather than direct predictors of STS.

Consistent with previous research, qualifications among interpreters were highly heterogeneous: About one third reported no formal training, a similar proportion held a university degree in interpreting, and the remainder had completed shorter workshop‐based or more extensive training programs (Hanft‐Robert and Mösko 2024; Wichmann et al. 2018). Despite this variability, interpreters demonstrated acceptable PTSD‐related knowledge and reported an average of almost 10 years of professional experience. Contrary to studies suggesting protective effects of training and experience (Geiling et al. 2021; Hanft‐Robert and Mösko 2024; Lerias and Byrne 2003), the present findings align with research reporting mixed or null effects of professional competence (Kindermann et al. 2017; Lai and Heydon 2015). This may reflect the absence of standardized training requirements and the heterogeneous professional contexts in which many ad hoc interpreters work, including medical, educational, asylum, social work and psychotherapeutic settings (Hanft‐Robert and Mösko 2024). In this context, the development of standardized nationwide training programs incorporating psychotherapy‐specific competencies appears warranted (Hapfelmeier et al. 2025).

Work‐related indicators suggest challenging and often unstable working conditions. Despite contributing more than 10 h of interpreting per week on average, monthly income was highly variable and generally low, indicating financial insecurity. Although about half of the interpreters reported access to supervision, the low number of supervision hours suggests that structured professional support remains limited, consistent with previous findings from Germany (Hanft‐Robert and Mösko 2024). At the same time, high ratings of relationship quality and perceived respect from therapists indicate largely positive collaboration within psychotherapeutic settings. Contrary to earlier studies suggesting that supervision, collaboration or workload influence STS (Denkinger et al. 2018; Geiling et al. 2021; Hanft‐Robert and Mösko 2024; Hapfelmeier et al. 2025), none of the assessed work‐related factors predicted STS in the present sample. This may indicate that occupational conditions play a smaller role than previously assumed or that their effects are overshadowed by more salient individual or contextual variables.

Daniels' (2006) model proposes that empathy, kindling processes and dissociation contribute to secondary traumatization by weakening self–other boundaries. In the present study, however, empathy showed no significant effect, consistent with mixed findings in the literature (Bridger et al. 2020; Püttker et al. 2015). Instead, identification with the patient's cultural background was associated with STS, suggesting that cultural alignment rather than empathic engagement may be more relevant in this context.

Overall, the absence of effects for general risk and protective factors may reflect the stronger influence of interpreter‐specific variables such as identification with clients. Furthermore, as noted by Hanft‐Robert and Mösko (2024), interpreters often work under poorly standardized conditions with limited training structures and support systems, particularly in psychotherapy settings where higher professional competence and support are required. Such conditions may have produced ceiling effects that contributed to the lack of statistical significance in the present analyses.

### 4.3 Interpreter Specific Risk Factors

In this sample, one third of the interpreters reported their own flight experience, consistent with previous findings (Geiling et al. 2022; Hapfelmeier et al. 2025; Kindermann et al. 2017). Our results suggest that personal flight experience contributes to STS. This contrasts with findings by Kindermann et al. (2017) and Hapfelmeier et al. (2025), who reported associations between refugee experience and posttraumatic stress disorder but not STS. Because the interpreters in the present sample worked specifically in psychotherapeutic settings rather than general community contexts, they were likely exposed to more trauma‐related content and explicit discussion of traumatic experiences. Such conditions may be particularly stressful for interpreters with their own history of flight and could explain the divergence from earlier findings.

Identification with the clients' cultural background emerged as the strongest predictor of STS. This finding aligns with research showing that cultural or biographical proximity can intensify emotional involvement and increase vulnerability (Fennig and Denov 2021; Green et al. 2012; Hsieh and Kramer 2012; Resera et al. 2015). These results suggest that identification, rather than empathy alone, may amplify the risk of empathic distress when interpreting traumatic content (Mehus and Becher 2016). Cultural and biographical similarity may therefore increase interpreters' vulnerability to STS beyond general psychological distress. Identification may evoke shared cultural trauma (Gartley and Due 2017; Miller et al. 2005; Simms et al. 2021) and contribute to role confusion and conflicting expectations from clients and therapists (Hassan and Blackwood 2021). These factors may pose a greater risk for STS than more general risk or protective factors and should therefore be considered in psychotherapy‐specific training and supervision for interpreters.

## 5 Limitations

This study has several limitations that should be considered when interpreting the findings. First, the relatively small final sample size limited statistical power. Although a larger number of interpreters initially participated, the analytic sample was substantially reduced because of exclusions, dropouts and listwise deletion to ensure comparable sample sizes across regression models. This reduction may have introduced selection bias and further constrained the robustness of the results.

Second, recruitment via an ad hoc online snowball procedure limits the representativeness of the sample. Moreover, the study focused exclusively on interpreters working in psychotherapeutic settings—a comparatively small professional group with limited availability—thereby restricting the generalizability of the findings to other interpreting contexts. Although this is one of the first quantitative studies specifically targeting interpreters in psychotherapy, not all participants may have been equally exposed to traumatic content. Consequently, the emotional strain associated with both the interpreted material and the therapeutic setting is likely to have been heterogeneous across the sample.

Third, all data were collected via self‐report measures administered online, which may be subject to reporting and recall biases. In addition, to maintain statistical power, several composite scores were used. While this approach reduced model complexity, it may have compromised construct validity by combining theoretically distinct dimensions. This limitation is particularly relevant for the occupational distress composite, as its underlying components showed relatively weak intercorrelations, suggesting that these constructs may not reflect a single coherent dimension.

Finally, the cross‐sectional design precludes any conclusions about causality or temporal relationships between risk factors and secondary traumatic stress.

## 6 Implications

Despite its limitations, a key strength of this study lies in the comprehensive assessment of general and interpreter‐specific factors associated with secondary traumatic stress (STS) using validated instruments. The findings indicate that interpreters with personal flight experience and strong identification with clients are particularly vulnerable to STS, highlighting the need for targeted preventive measures in interpreter‐mediated psychotherapy.

These vulnerabilities may be more pronounced than in other professional groups, as such biographical and cultural proximity is typically not characteristic of clinicians or other psychosocial service providers (Miller et al. 2005). Structured support systems—including regular supervision, trauma‐informed and psychotherapy‐specific training and clear role clarification—therefore appear essential. This is particularly relevant for ad hoc interpreters, who often lack formal training, structured preparation and institutional support while being exposed to emotionally demanding content and culturally divergent role expectations (Miller et al. 2005; Hanft‐Robert and Mösko 2024). Overall, the findings underscore the importance of conceptualizing interpreters as a distinct professional group with specific risk profiles and point to an urgent need for further research on training, organizational support and preventive interventions tailored to this vulnerable population.

## Author Contributions

M.H. conceptualized and designed the study, conducted the data collection, performed data analysis and interpretation, and wrote the original draft of the manuscript. B.S. performed the statistical analyses and contributed to data interpretation. R.R. contributed to study design and manuscript revision. All authors contributed to manuscript revision, read, and approved the submitted version.

## Ethics Statement

The Institutional Review Board of the Catholic University Eichstaett‐Ingolstadt approved the research protocol. Written informed consent was obtained from all participants.

## Supporting information


**Appendix A** Assumptions of Regression Analysis.
**Figure B1** Partial‐residual plots.
**Figure B2** Q‐Q plot.
**Figure B3** Residuals versus fitted plot.
**Table B1** Variance inflation factor and tolerance of predictors.
Appendix B: Correlation Matrix.
Appendix Table C: Sensitivity Analyses Excluding Highly Correlated Predictors.

## Data Availability

The data that support the findings of this study are available on request from the corresponding author. The data are not publicly available due to privacy or ethical restrictions.
