# Supplementary material for: Secondary Traumatic Stress of Interpreters Working in Psychotherapy: Protective, Risk and Interpreter‐Specific Factors
Source: Clin Psychol Psychother. 2026 Jul 7;33(4):e70305. doi: 10.1002/cpp.70305 (PMC13340473; doi:10.1002/cpp.70305)
Supplement: Supplementary file 1 — Appendix A Assumptions of Regression Analysis. Figure B1 Partial‐residual plots. Figure B2 Q‐Q plot. Figure B3 Residuals versus fitted plot. Table B1 Variance inflation factor and tolerance of predictors. Appendix B: Correlation Matrix. Appendix Table C: Sensitivity Analyses Excluding Highly Correlated Predictors. [file CPP-33-e70305-s001.docx]

**Appendix**

[Appendix A: Assumptions of Regression Analysis 2](#_Toc230687058)

[Appendix B: Correlation matrix 5](#_Toc230687059)

[Appendix Table C: Sensitivity analyses excluding highly correlated predictors 7](#_Toc230687060)

# Appendix A: Assumptions of Regression Analysis

**Linearity**

**Figure B.1** Partial-Residual-Plots

**
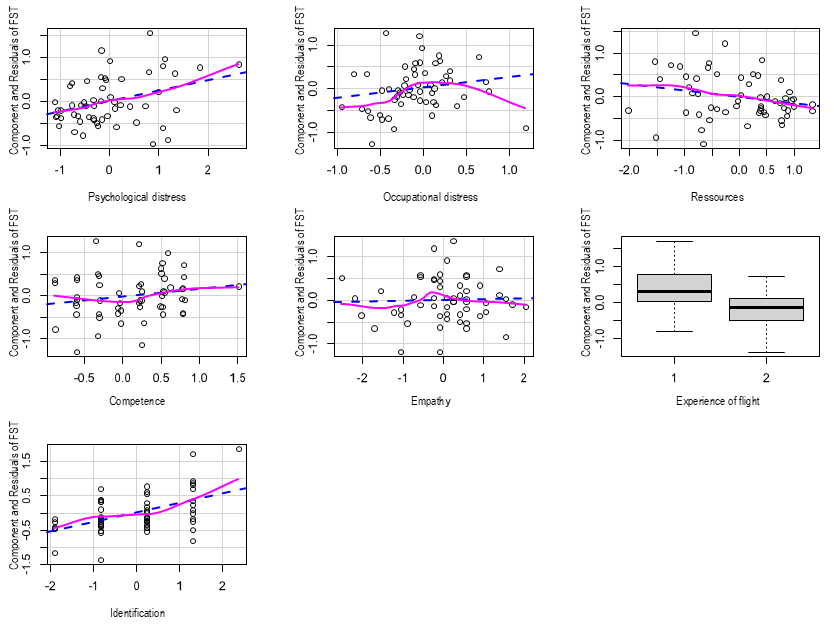
**

*Note*. FST = Secondary traumatic stress.

**Normal distribution of residuals**

**Figure B.2** Q-Q Plot

**
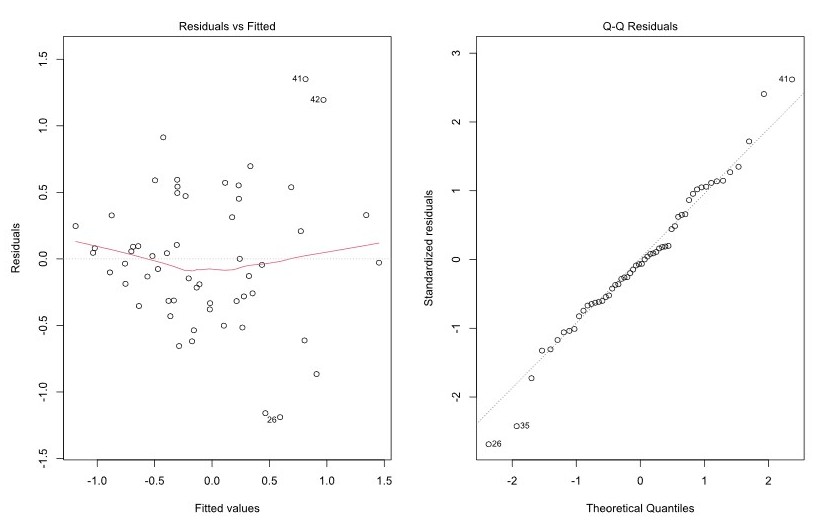
**

Shapiro Wilk Test: *W* = 0.98, *p* = 0.652

**Homoscedasticity**

**Figure B.3** Residuals vs. Fitted Plot

**
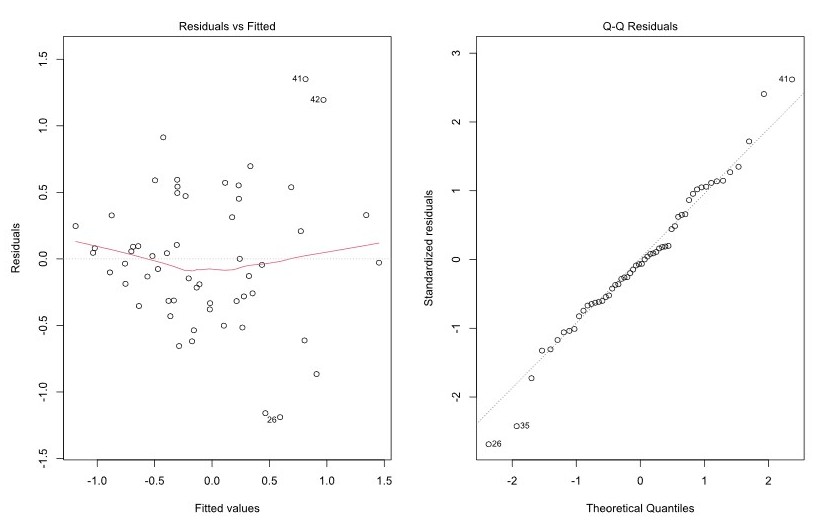
**

Breusch Pagan Test: *χ*²(7) = 14.09, *p* = .050

**Multicolinearity**

**Table B.1** Variance Inflation Factor and Tolerance of Predictors

| Predictor | VIF | Tolerance |
| --- | --- | --- |
| Psychological distress | 2.26 | 0.44 |
| Occupational distress | 1.06 | 0.94 |
| Ressources | 2.20 | 0.45 |
| Competence | 1.20 | 0.83 |
| Empathy | 1.42 | 0.70 |
| Flight experience | 1.21 | 0.82 |
| Identification | 1.38 | 0.72 |

# Appendix B: Correlation matrix

| Variable | 1 | 2 | 3 | 4 | 5 | 6 | 7 | 8 | 9 | 10 | 11 | 12 | 13 | 14 |
| --- | --- | --- | --- | --- | --- | --- | --- | --- | --- | --- | --- | --- | --- | --- |
| 1. Secondary stress |  |  |  |  |  |  |  |  |  |  |  |  |  |  |
|  |  |  |  |  |  |  |  |  |  |  |  |  |  |  |
| 2. PCL | .76** |  |  |  |  |  |  |  |  |  |  |  |  |  |
|  | [.41, .92] |  |  |  |  |  |  |  |  |  |  |  |  |  |
|  |  |  |  |  |  |  |  |  |  |  |  |  |  |  |
| 3. PHQ | .83** | .82** |  |  |  |  |  |  |  |  |  |  |  |  |
|  | [.55, .94] | [.53, .94] |  |  |  |  |  |  |  |  |  |  |  |  |
|  |  |  |  |  |  |  |  |  |  |  |  |  |  |  |
| 4. GAD | .76** | .83** | .87** |  |  |  |  |  |  |  |  |  |  |  |
|  | [.40, .91] | [.56, .94] | [.64, .95] |  |  |  |  |  |  |  |  |  |  |  |
|  |  |  |  |  |  |  |  |  |  |  |  |  |  |  |
| 5. Hours | .24 | .12 | .13 | .11 |  |  |  |  |  |  |  |  |  |  |
|  | [-.31, .67] | [-.42, .60] | [-.41, .60] | [-.43, .59] |  |  |  |  |  |  |  |  |  |  |
|  |  |  |  |  |  |  |  |  |  |  |  |  |  |  |
| 6. Professional support | -.40 | -.17 | -.10 | -.29 | -.41 |  |  |  |  |  |  |  |  |  |
|  | [-.76, .14] | [-.63, .38] | [-.59, .43] | [-.70, .26] | [-.76, .12] |  |  |  |  |  |  |  |  |  |
|  |  |  |  |  |  |  |  |  |  |  |  |  |  |  |
| 7. Salary | .10 | -.09 | -.06 | -.06 | .84** | -.33 |  |  |  |  |  |  |  |  |
|  | [-.44, .58] | [-.58, .44] | [-.55, .47] | [-.56, .47] | [.57, .94] | [-.72, .22] |  |  |  |  |  |  |  |  |
|  |  |  |  |  |  |  |  |  |  |  |  |  |  |  |
| 8. Coherence | -.85** | -.91** | -.96** | -.91** | -.11 | .12 | .07 |  |  |  |  |  |  |  |
|  | [-.95, -.60] | [-.97, -.75] | [-.99, -.88] | [-.97, -.75] | [-.59, .43] | [-.42, .60] | [-.46, .56] |  |  |  |  |  |  |  |
|  |  |  |  |  |  |  |  |  |  |  |  |  |  |  |
| 9. Social support | -.76** | -.79** | -.86** | -.79** | -.13 | .26 | .07 | .87** |  |  |  |  |  |  |
|  | [-.91, -.40] | [-.93, -.48] | [-.95, -.61] | [-.93, -.47] | [-.60, .41] | [-.29, .68] | [-.46, .56] | [.64, .96] |  |  |  |  |  |  |
|  |  |  |  |  |  |  |  |  |  |  |  |  |  |  |
| 10. Qualification | .14 | .04 | .14 | .09 | .16 | .07 | .40 | -.11 | -.06 |  |  |  |  |  |
|  | [-.40, .61] | [-.49, .54] | [-.40, .61] | [-.44, .58] | [-.38, .62] | [-.46, .56] | [-.14, .76] | [-.59, .43] | [-.56, .46] |  |  |  |  |  |
|  |  |  |  |  |  |  |  |  |  |  |  |  |  |  |
| 11. Experience | .54* | .36 | .30 | .48 | -.02 | -.53* | -.19 | -.43 | -.40 | -.26 |  |  |  |  |
|  | [.03, .82] | [-.19, .73] | [-.25, .70] | [-.04, .80] | [-.53, .49] | [-.82, -.02] | [-.64, .35] | [-.77, .11] | [-.76, .14] | [-.68, .30] |  |  |  |  |
|  |  |  |  |  |  |  |  |  |  |  |  |  |  |  |
| 12. Knowledge | -.78** | -.56* | -.55* | -.61* | -.20 | .33 | .06 | .63* | .53* | .26 | -.71** |  |  |  |
|  | [-.93, -.46] | [-.84, -.07] | [-.83, -.05] | [-.85, -.14] | [-.65, .35] | [-.22, .72] | [-.47, .55] | [.17, .86] | [.02, .82] | [-.29, .68] | [-.89, -.30] |  |  |  |
|  |  |  |  |  |  |  |  |  |  |  |  |  |  |  |
| 13. Empathy | -.07 | -.04 | -.03 | -.04 | -.13 | .16 | .03 | .03 | .26 | .30 | -.16 | .30 |  |  |
|  | [-.56, .46] | [-.54, .48] | [-.53, .49] | [-.54, .48] | [-.60, .41] | [-.38, .62] | [-.49, .54] | [-.49, .54] | [-.29, .68] | [-.25, .71] | [-.62, .39] | [-.25, .70] |  |  |
|  |  |  |  |  |  |  |  |  |  |  |  |  |  |  |
| 14. Expierience of flight | -.79** | -.51 | -.52* | -.39 | -.17 | .29 | -.00 | .54* | .50 | .20 | -.56* | .78** | .23 |  |
|  | [-.93, -.46] | [-.81, .00] | [-.82, -.01] | [-.75, .16] | [-.63, .37] | [-.26, .70] | [-.51, .51] | [.03, .82] | [-.02, .80] | [-.35, .65] | [-.83, -.07] | [.44, .92] | [-.32, .66] |  |
|  |  |  |  |  |  |  |  |  |  |  |  |  |  |  |
| 15. Identity | .85** | .69** | .67** | .61* | .13 | -.13 | .00 | -.74** | -.53* | .16 | .43 | -.75** | .12 | -.69** |
|  | [.60, .95] | [.27, .89] | [.25, .88] | [.14, .85] | [-.41, .61] | [-.60, .41] | [-.51, .52] | [-.91, -.36] | [-.82, -.03] | [-.39, .62] | [-.11, .77] | [-.91, -.39] | [-.41, .60] | [-.89, -.28] |

*Note.* Values in square brackets indicate the 95% confidence interval for each correlation. * indicates *p* < .05. ** indicates *p* < .01.

# Appendix Table C: Sensitivity analyses excluding highly correlated predictors

| Predictor | β Full model | β Without ressources | β Without psychological distress |
| --- | --- | --- | --- |
| Intercept | **0.30** | **0.33 *** | 0.27 |
| Psychological distresss | 0.24 | **0.34**** | — |
| Occupational distress | 0.23 | 0.24 | 0.24 |
| Ressources | -0.15 | — | **−0.31**** |
| Competence | 0.17 | 0.16 | 0.21 |
| Empathy | 0.19 | −0.02 | 0.05 |
| Flight experience | **0.54**** | **0.56**** | **0.49**** |
| Identification | **0.27**** | **0.27**** | **0.31***** |
| Adjusted R² | .50 | .52 | .48 |

Note. Unstandardized regression coefficients are reported. Psychological distress and resources were analyzed separately due to their high correlation (r = .97). * p < .05. ** p < .01. *** p < .001.
